# Grain Yield Response of Corn (*Zea mays* L.) to Nitrogen Management Practices and Flooding

**DOI:** 10.3390/plants9030348

**Published:** 2020-03-10

**Authors:** Taylor E. Dill, Steven K. Harrison, Steven W. Culman, Alexander J. Lindsey

**Affiliations:** 1 Department of Horticulture and Crop Science, The Ohio State University, 2021 Coffey Rd., Columbus, OH 43210, USA; dill.138@buckeyemail.osu.edu (T.E.D.); harrison.9@osu.edu (S.K.H.); 2 School of Environment and Natural Resources, The Ohio State University, 1680 Madison Ave., Wooster, OH 44691, USA; culman.2@osu.edu

**Keywords:** corn, nitrogen, flooding, flood, sidedress, yield

## Abstract

Flooding can reduce corn growth and yield, but nitrogen (N) management practices may alter the degree to which plants are negatively impacted. Damage caused by flooded conditions may also affect the utilization of a post-flood N application to increase yield. The objectives of this study were to evaluate how pre-plant and pre-plant plus post-flood N applications contribute to corn growth and yield following flood conditions and to quantify the partial return of employing different N management strategies in the event of a flood. A field study was conducted in Ohio using four flood durations (FD; 0, 2, 4, or 6 days initiated at V4 to V5) and three N management practices (0 kg N ha^−1^, 134 kg N ha^−1^ applied pre-plant, and 134 pre-plant + 67 kg N ha^−1^ applied post-flooding). Application of 134 kg N ha^−1^ increased yield compared to 0 kg N ha^−1^ by 65%, 68%, 43% and 16% for 0 d, 2 d, 4 d, and 6 d FD, respectively; the application of 134 + 67 kg N ha^−1^ increased grain yield compared to 134 kg N ha^−1^ by 7%, 27%, 70%, or 55% for 0 d, 2 d, 4 d, or 6 d FD, respectively. Partial return analysis produced similar results to those for grain yield. Results suggest that in regions prone to early-season flooding, additional N applied post-flood can improve yield and partial return compared to the application of pre-plant alone at a lower rate or no N. Results indicate that total soil nitrate-N levels two weeks after flood initiation may serve as a good predictor of yield.

## 1. Introduction

In the Midwestern and Northeastern United States, the frequency of extreme weather events and total precipitation has been increasing since 1910 by 1% to 3% each decade [1]. Winter and spring precipitation, as well as the occurrence of heavy downpours, is anticipated to increase more for the northern United States than the Southeastern and Southwestern regions [2]. Heavy downpours are classified as the amount of precipitation in the >95th percentile of daily precipitation [1]. The quantity of rain that is falling on the days of the heaviest precipitation has been increasing [2], particularly in the Midwest, where 30% to 50% of total annual precipitation occurs on the ten wettest days of the year, mostly in the spring [3]. Because heavy rain events are increasing along with the amount of rain within those events [4], the frequency of flooding in agricultural fields is likely to increase.

Excessive precipitation can inhibit seed germination and emergence, and affect vegetative growth of corn. Flooding can reduce corn height, biomass production [5,6,7], yield [5,8,9], and photosynthetic efficiency by damaging leaf chloroplasts [10]. Excessive rainfall can reduce corn yield by up to 34% compared to the expected yield, which is similar to the 37% reduction observed from extreme drought stress [11].

Flooded conditions can impact N application timing and efficiency as well as the dominant form of N in the soil at any given time [12]. Nitrogen management in corn is a key factor for producers to consider as the mineralization rate in many soils is insufficient to meet crop yield goals. However, N is a dynamic element in the soil due to its interaction with both chemical (i.e., ammonium content on soil exchange sites; nitrate (NO_3_) ions moving in soil solution) and biological factors (i.e., nitrification process, denitrification process). Nitrogen is lost through denitrification and leaching in flooded or saturated soils. Gaseous losses due to denitrification have been reported as 0%–20% of fertilizer N applied, but annual losses were 50%−120% greater in undrained soils compared to drained soils [13]. Soil NO_3_ content in drainage water has also been shown to increase with N application and source, ranging from 21 to 84 kg NO_3_-N ha^−1^ in a continuous corn cropping system grown on silt loam soil [14]. Other researchers have reported that 15% of applied N is deposited into groundwater, with levels increasing in years with above-normal precipitation [15]. Due to the impact of soil water content on chemical N transformations and movement, understanding how N management can impact corn growth and recovery in the event of a flood is critical for efficient nutrient management and ultimately a profitable operation. 

Current recommendations in the Eastern U.S. Corn Belt for corn grown in environments at risk for N loss are to apply N at two stages, which can be referred to as a split application. The first application should occur within six weeks of planting, and the second (or sidedress) application should occur within 10 weeks of planting [16]. Even where a lower N rate was applied pre-plant (with the intention to sidedress the remainder of the planned N application rate), producers often question the viability of applying sidedress N following a flood event. Current crop assessments for in-season N adjustment typically occur in three stages: when corn is 6−12 inches tall (pre-sidedress nitrate test) as a soil test [17], leaf tissue analysis at VT (tasseling) to R1 (silking) [18], or as a stalk nitrate test after R6 (physiological maturity) [19] (staging method described by Abendroth et al. [20]). However, a flood event occurring between V4 and V5 may require an in-season assessment outside the typical sampling time for implementing a post-flood nutrient application.

Past research has indicated supplemental N application post-flood can reduce the yield penalty if flooding occurs [5,7,8], but the response may differ from past research under soil and weather conditions typical of the Upper Midwestern United States. The duration of flooding may affect the response to a post-flood N application. In a recent study from Missouri, a rescue N application of 84 kg N ha^−1^ was effective at increasing grain yield for a 3-day flood initiated at V6, but the benefit was not seen following a 7-day flood [7]. It is unclear how well damaged plants would be able to utilize a post-flood N application in new environments, or whether economic returns would be sufficient to justify the second N application for producers implementing a split N application approach. The objectives of this study were to evaluate how pre-plant and pre-plant plus post-flood N applications impact corn phenology and grain yield in the event of flooding and to quantify the partial return of employing different N management strategies in the event of varied flooding conditions.

## 2. Results

### 2.1. Weather Conditions 

Average air temperatures during the 2017 growing season at the Waterman Agricultural and Natural Resources Laboratory (WNRL) were within 5% of the 30-year average (1989–2018) for all months except October, where temperatures were 19% greater than the 30-year average (Table 1). The average monthly precipitation at WNRL was greater than the 30-year average in May, July, and October by 18% to 80%. Conversely, June, August, and September had less rain than the 30-year average by 11% to 50%. The 2018 growing season temperatures were within 13% of the 30-year average for all months except May (Table 1), where temperature was 24% to 28% higher than the 30-year average. The precipitation in June and August of 2018 was greater than the 30-year average, ranging from 16% to 60% above average. Despite the flood treatments being initiated in late-June at WRNL in 2017, and in late-May (WRNL) or early-June (Western Agricultural Research Station or WARS) in 2018, the average daily maximum and daily average temperatures during and after flooding were within one degree C in each environment (Table 2). The frequency of rainfall after flooding was terminated (at least one event every three days) helped ensure that broadcast N treatments applied post-flooding were incorporated into the soil.

### 2.2. Flooding and N Impacts on Crop Phenology and Growth 

On 0 days after flood initiation (DAFI), average vegetative growth stage (4.2–4.3), height (42.9–45.6 cm), and leaf greenness (37.7–41.0 SPAD value) were similar across all treatments (*p* > 0.05; Table S1). On 14 DAFI, vegetative growth stage was less (*p* < 0.001) for the 4 d and 6 d FD by 1.0−1.1 stages compared to the 0 d FD across NMP (vegetative growth stages of 6.6, 6.5, and 7.6, respectively), but the 2 d FD exhibited a similar growth stage (7.3) to the 0 d FD. The vegetative growth stage of plants receiving 0 kg N ha^−1^ (across FD) was 0.5 less (*p* < 0.001) compared to when 134 or 134 + 67 kg N ha^−1^ was applied (vegetative growth stages of 6.6, 7.1 and 7.1, respectively).

**Figure 1:**
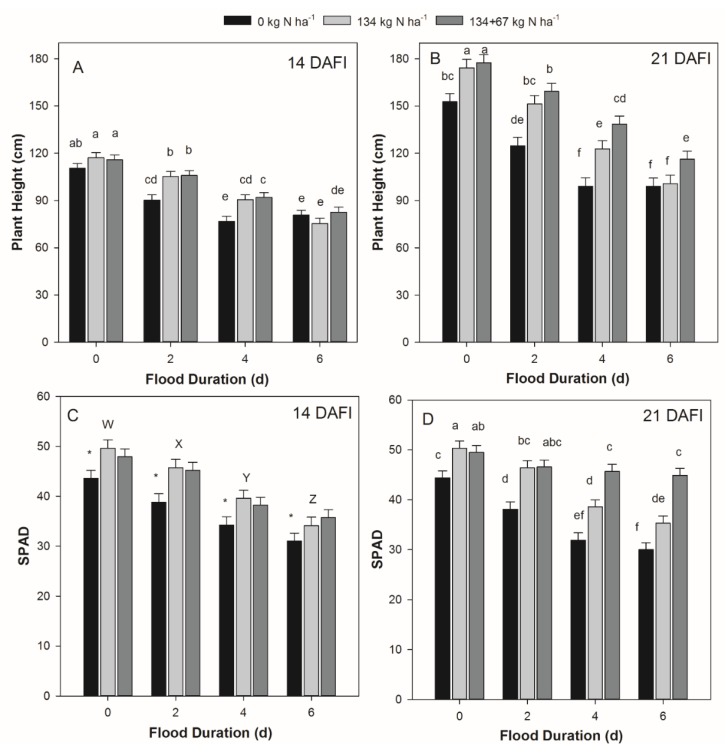
(**A**) Plant height 14 days after flood initiation (DAFI), (**B**) plant height 21 DAFI, (**C**) leaf greenness 14 DAFI, and (**D**) leaf greenness 21 DAFI for each flood duration (FD) and N application rate (NMP) of 0, 134, or 134 + 67 kg N ha^−1^. Error bars denote standard error of the mean. When present, different lowercase letters within a panel indicate differences in means for the FD × NMP interaction. Uppercase letters denote differences in FD across NMP, and an asterisk denotes a significantly lower mean for the specified NMP (across FD) when the FD × NMP interaction was non-significant.

On 14 DAFI, an FD × NMP interaction (*p* = 0.019) was present for plant height (Figure 1A). Heights were similar across NMP within the 0 d and 6 d FD, but plants under 6 d FD were 30% shorter than the plants under 0 d FD. The lack of pre-plant N (0 kg N ha^−1^) resulted in 15% shorter plants than the other N management treatments under 2 d and 4 d FD (Figure 1A). The interaction of FD × NMP for height 21 DAFI (*p* = 0.049) showed a height reduction in the absence of pre-plant N for all FD, but heights were similar between 134 and 134 + 67 kg N ha^−1^ within the 0 d FD and within the 2 d FD. This suggests soil N content may have been adequate to facilitate growth following a 2 d FD, or that plant damage was minimal and allowed for quicker resumption of growth compared to the other FD treatments (Figure 1B). In the 4 d FD when 134 kg N ha^−1^ was applied pre-plant, height was 24% greater compared to 0 kg N ha^−1^ and height for 134 + 67 kg N ha^−1^ was 12% greater than 134 kg N ha^−1^. In the 6 d FD, height was similar for the 0 and 134 kg N ha^−1^ treatments and was 14% less than height of plants in the 134 + 67 kg N ha^−1^ treatment. 

In 2018, shoot biomass and root biomass, length, area, and porosity were similar across treatments at 0 DAFI (Table 3). Shoot biomass decreased with FD by 14 DAFI and was less when 0 kg N ha^−1^ was applied compared to 134 or 134 + 67 kg N ha^−1^. Root biomass was similar between treatments 14 DAFI, but root porosity at 14 DAFI increased with FD by 30% to 50% compared to the 0 d FD, indicating that root tissue may have formed aerenchyma as a response to the earlier flooded conditions.

In the current study, leaf greenness (as quantified through SPAD value) was reduced 14 DAFI from the 0 d FD by 11%, 21%, or 30% for the 2 d, 4 d, or 6 d FD, respectively (*p* < 0.0001; Figure 1C). The 0 kg N ha^−1^ treatment also had a 16% lower SPAD value than the 134 and 134 + 67 kg N ha^−1^ treatments (*p* < 0.001; Figure 1C). The SPAD value interaction of FD × NMP 21 DAFI (*p* < 0.001) may indicate that uptake of the post-flood N application occurred in the 2 d and 4 d FD or improved remobilization of existing N reserves within the plant, with increases in SPAD value ranging from 18% to 50% (Figure 1D) compared to the 0 and 134 kg N ha^−1^ treatments. 

Ear-leaf N concentration at R1 was similar in the 0 d FD when 134 or 134 + 67 kg N ha^−1^ was applied, but when flooding occurred the ear-leaf N concentrations were 14% to 23% less with the 134 kg N ha^−1^ than with 134 + 67 kg N ha^−1^ (Table 4). However, the 2 d FD ear-leaf N concentration was 12% or 14% greater than the 4 d and 6 d FD, respectively, when 134 kg N ha^−1^ was applied, suggesting that a 2 d FD may experience less soil N loss or less damage to roots, or a combination of these, to facilitate greater uptake prior to R1.

The R1 total soil NO_3_-N results were significant for FD, NMP and FD x NMP (Table 4). The FD × NMP interaction was caused by greater total soil NO_3_-N in 0 d 134 + 67 kg N ha^−1^ compared to all other treatments, which may suggest that total soil NO_3_-N supply exceeded plant demand in this treatment.

### 2.3. Corn Grain Yield, Moisture, and Partial Return 

Corn grain yields were similar for 0 d FD that received 134 or 134 + 67 kg N ha^−1^, but the yield gained from the 67 kg N ha^−1^ post-flood N application of ranged from 27% to 70% in all other FD compared to the yield in the 134 kg N ha^−1^ treatment (Table 4). Grain yield of the 2 d FD with 134 + 67 kg N ha^−1^ was similar to 0 d FD with 134 and 134 + 67 kg N ha^−1^ (Table 4). Yield of the 2 d and 4 d FD that received 134 kg N ha^−1^ were 68% or 42% greater, respectively, than the same FD that received 0 kg N ha^−1^. However, minimal difference in yield was observed between 0 and 134 kg N ha^−1^ application for the 6 d FD. Across NMP, a linear relationship between FD and yield was observed (*p* < 0.0001). For each additional day of flooding, yield was reduced by 870 kg ha^−1^. Grain moisture measured on the day of harvest was not significantly different based on FD, NMP or FD × NMP, and ranged from 190 to 200 g kg^−1^ (Table S2). Stalk lodging was less than 5% for all treatments (Table S2).

The partial return assessment mirrored grain yield results closely, where the greatest return was observed for the 0 d FD receiving 134 or 134 + 67 kg N ha^−1^, followed by the 2 d FD with 134 + 67 kg N ha^−1^ (Table 4). The partial return for a 4 d FD receiving 134 + 67 kg N ha^−1^ was 73% greater than with 134 kg N ha^−1^ alone. Additionally, the 4 d FD with 134 kg N ha^−1^ applied produced 26% greater partial return than the 4 d FD receiving 0 kg N ha^−1^. Although the 6 d FD with 134 kg N ha^−1^ produced 1000 kg ha^−1^ greater yield than the 6 d FD with 0 kg N ha^−1^, the partial return was only $5 ha^−1^ greater. The 6 d FD with 134 + 67 kg N ha^−1^ partial return exceeded the 6 d FD with 134 kg N ha^−1^ by 55%. The application of N post-flood appeared to be justifiable economically for all FDs investigated in this study.

The NUE (yield gained from unit N applied) changed with FD as well (Table 5). The NUE of applying 134 kg N ha^−1^ (yield gain over 0 kg N ha^−1^ treatments) was greatest under 0 or 2 d FD, but a positive NUE value was still observed for the 4 d FD. The 6 d FD resulted in the lowest NUE, and the value of 5.31 was similar to 0 (*p* = 0.306). The NUE for the 67 kg N ha^−1^ post-flood application (yield gained over 134 kg N ha^−1^ treatment) was greatest for the 4 and 6 d FD. Additionally, in this study, the NUE for the 0 d FD from the 134 + 67 NMP was also not different from 0 (*p* = 0.171). 

These results suggest that a pre-plant N application improved yield and partial economic return up to the 4 d FD, but the pre-plant N application resulted in minimal differences following the 6 d FD. Regardless of FD, N applied post-flood helped increase yield and partial economic return. Plants under longer FD exhibited greater NUE for the post-flood application. 

### 2.4. Post-Flood Total Soil NO_3_-N Concentrations

In 2018, the total soil NO_3_-N samples collected on 0 and 14 DAFI help to explain the fate of early-season total soil NO_3_-N post-flood (Table 6). On 0 DAFI, only NMP was significant (*p* < 0.001) for total soil NO_3_-N due to the detection of the pre-plant N that was incorporated into the soil. On 14 DAFI, which was after the post-flood N application to each FD, the effects of FD, NMP and FD × NMP were significant (Table 6). By 14 DAFI, the 2 d, 4 d, and 6 d FD treatments exhibited total soil NO_3_-N levels similar in most cases to the 0 kg N ha^−1^ 0 d FD suggesting that substantial loss of the pre-plant N from the upper soil profile was experienced. The 0 d FD with 134 + 67 kg N ha^−1^ had the highest NO_3_-N concentrations, and the 134 + 67 kg N ha^−1^ with 2 d and 4 d FD were similar to the concentrations of the 0 d FD with 134 kg N ha^−1^. The total soil NO_3_-N values collected 14 DAFI were almost identical in rank order (and statistical separation) as the ear-leaf N concentration results from 2018 (Table 6).

**Figure 2:**
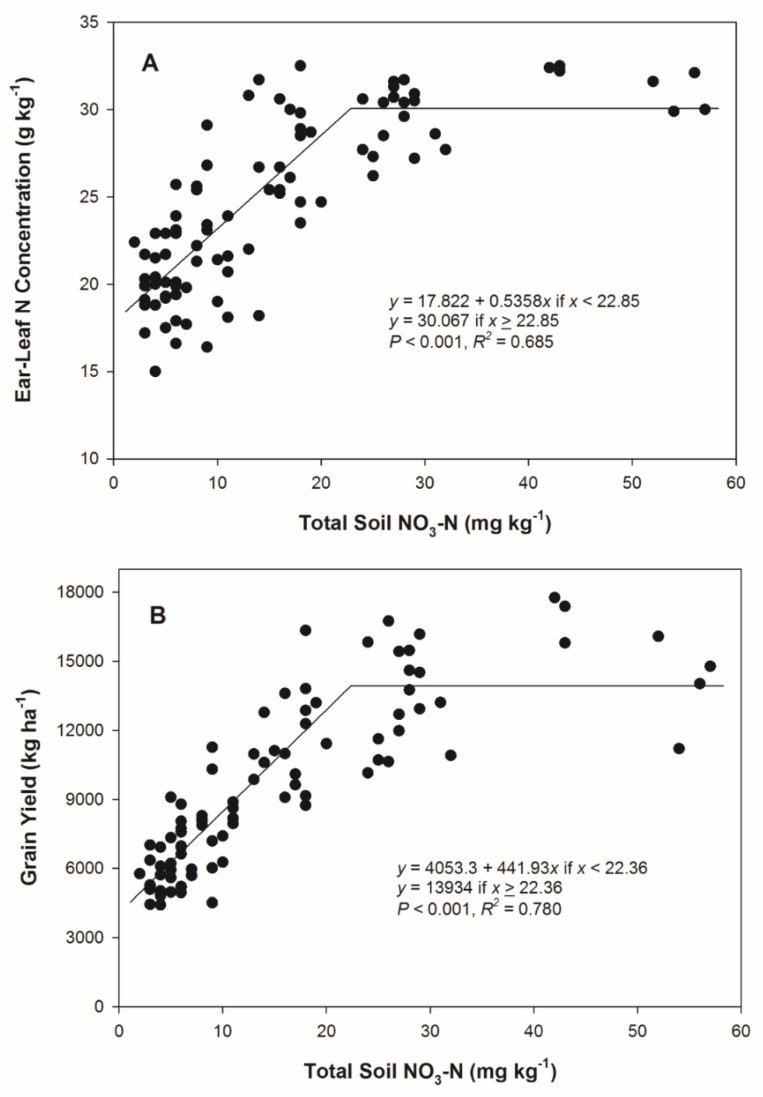
(**A**) Ear-leaf N concentration at R1 as impacted by soil nitrate-nitrogen (NO_3_-N) 14 days after flood initiation in 2018. (**B**) Grain yield as impacted by total soil NO_3_-N 14 days after flood initiation in 2018.

Identification of N deficiency as a result of flooded conditions in corn prior to R1 would enable producers to apply a rescue N application post-flood to reduce the likelihood of grain yield loss. The 14 DAFI total soil NO_3_-N level was regressed on ear-leaf N concentration at R1 and grain yield from 2018 to assess if a post-flood soil test may help predict either of these parameters (Figure 2). A linear–plateau relationship was evident, with an increase of 0.54 g kg^−1^ ear-leaf N for every 1.0 mg kg^−1^ increase in total soil NO_3_-N and a plateau appearing once total soil NO_3_-N exceeded 22.9 mg kg^−1^ (*r^2^* = 0.685, Figure 2A). This was similar to the yield response to increasing total soil NO_3_-N, where the plateau in yield was achieved at 22.4 mg kg^−1^ total soil NO_3_-N (*r^2^* = 0.780, Figure 2B).

**Figure 3:**
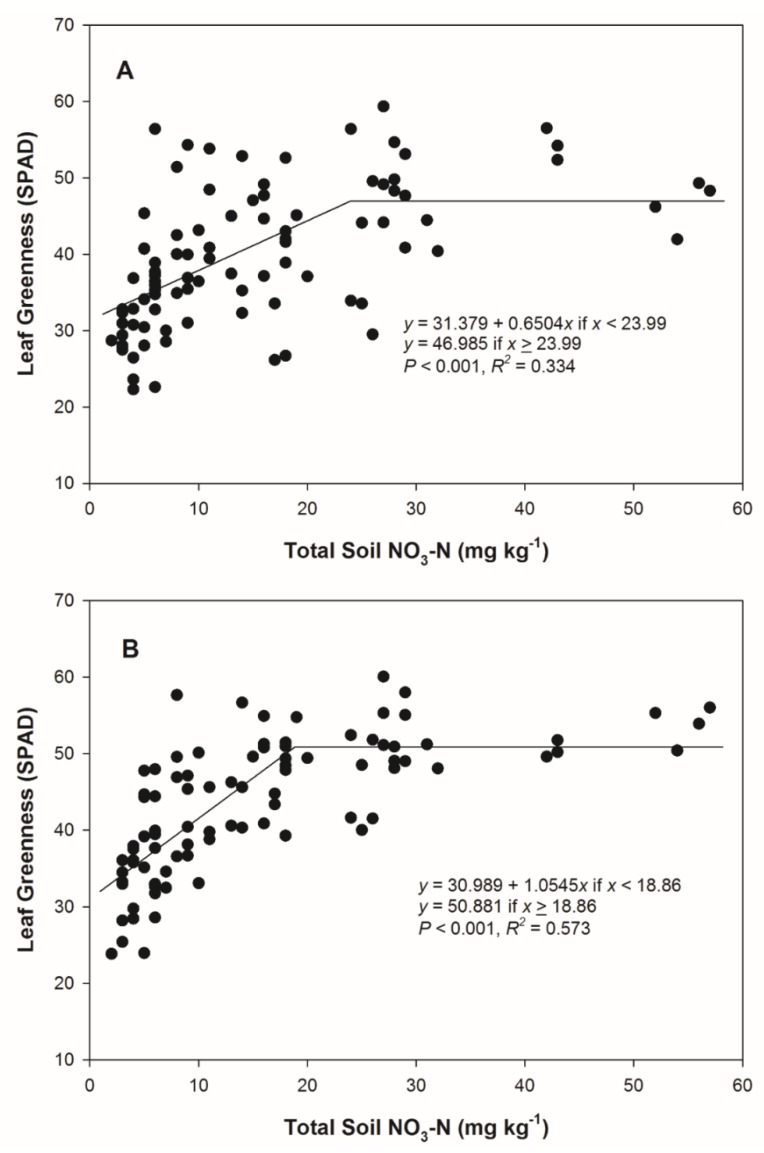
(**A**) Leaf greenness (as SPAD) 14 days after flood initiation (DAFI) as impacted by soil nitrate-nitrogen (NO_3_-N) level 14 DAFI in 2018. (**B**) Leaf greenness (as SPAD) 21 DAFI as impacted by total soil NO_3_-N level 14 DAFI in 2018.

A possible alternative to soil sampling to assess corn N status post-flood may be the use of a leaf-greenness sensor, as SPAD values have corresponded to differences in N application after the V6 growth stage in Ohio and Michigan [21]. The leaf greenness, as impacted by total soil NO_3_-N 14 DAFI, was explained with a linear–plateau relationship in the current study (Figure 3). Leaf greenness at 14 and 21 DAFI increased until total soil NO_3_-N values exceeded 24.0 and 18.9 mg kg^−1^, respectively. However, *r^2^* values for these models were 0.334 and 0.573, respectively.

## 3. Discussion

Reduced plant height and lower vegetative growth stage values under longer FD may have been due greater physiological stress in addition to changes in the amount of available soil N due to leaching and denitrification [7,22]. Morphological changes related to aerenchyma formation to facilitate oxygen transfer to root apical tissue may alter future plant growth patterns [23]. Total root area 14 DAFI increased with FD and decreasing N application rates, suggesting greater root exploration to escape flood conditions or increase nutrient capture (Table 3). This was further supported by the increased root porosity without changing root biomass. Plants were responding morphologically to the flooded conditions in this experiment, and this response may impact the degree to which plants can utilize a post-flood N application if applied.

Partial submergence and waterlogging can induce physiological changes in plants related to leaf water potential, photosynthetic rates, and phloem transport of sugars [23]. Reduced N uptake from the soil solution can also contribute to the decimation of chlorophyll and chloroplasts [10], which could cause leaves to alter their greenness and light absorption efficiency. The SPAD value interaction of FD × NMP 21 DAFI (Figure 1D) may indicate that uptake of the applied post-flood N in the 2 d and 4 d FD or improved remobilization of existing N reserves within the plant compared to the other NMP. Other studies reported an increase in leaf chlorophyll content or SPAD value with increased N application rate [21,24]. The reduction in ear-leaf N concentration with increased flood duration is likely due in part to losses of N by denitrification and NO_3_ leaching [7,22]. It is unlikely that these results were due to dilution within the tissue, because increasing FD and decreasing N application also decreased ear-leaf biomass (Table S3). The treatments exhibiting ear-leaf N concentration greater than 29.0 g kg^−1^ were within the sufficiency range (29.0–35.0 g kg^−1^) to prevent yield reduction due to N deficiency [16]. The R1 ear-leaf N concentration showed that the post-flood N application of 67 kg N ha^−1^ for 4 d and 6 d FD may have been insufficient to prevent N deficiency (ear-leaf N concentration less than 29.0 g kg^−1^). However, it is unclear whether changes in root characteristics, as observed in 2018, would have prevented the plant from absorbing additional N had it been applied to the soil given multiple post-flood N rates were not implemented in this study.

The yield loss per day of flood was 870 kg ha^−1^, which is greater than reported in past research (420–720 kg ha^−1^ decrease in yield per day) when flooding occurred at the V6 growth stage [7]. Corn becomes more tolerant to flooding as growth stage advances, as flooding at 15 cm height resulted in greater yield loss per day (410–608 kg ha^−1^) than flooding at 76 cm (203–337 kg ha^−1^) [8]. There are conflicting ideologies regarding the split application of N, with some authors generally stating that its effectiveness depends on timing and precipitation [25,26,27]. Past researchers have also observed varied yield increases with N application in the event of increasing FD or precipitation [7,8,25]. The current study results suggest that a reduction in total soil NO_3_-N 14 DAFI contributed to yield loss when FD exceeded two days. However, the pre-plant application of 134 kg N ha^−1^ increased yield for both the 4 d and 6 d FD compared to 0 kg N ha^−1^ within each FD, which may suggest that early-season plant uptake or use contributed to the yield increase. 

Because pre-plant N loss can occur with flood events, the potential for rescue N applications post-flood should be considered. Given that the total soil NO_3_-N in the 4 d FD 14 DAFI was similar regardless of whether a pre-plant N application of 134 kg N ha^−1^ was made, producers may benefit economically from delaying a portion of the planned N for application if early-vegetative flooding is anticipated. The post-flood N application in the current study was made between growth stages V6 and V7, which is within the recommended time frame for a split application practice [16]. Scharf et al. [28] observed N applications through V11 can result in minimal yield loss compared to pre-plant applications. Evaluation of total soil NO_3_-N within a week after a flood event ending would afford the producer time to assess soil N status and apply a rescue N treatment if warranted. Further work on the timing of N application with flooded conditions and calculating in-season N application based on post-flood measurements should be conducted.

A post-flood N application was an economically viable option for reducing grain yield losses in the event of a 2 to 6 d FD under the conditions of the current study. The NUE observed in this study suggests efficiency of applied fertilizer pre-plant decreased with flooding, but even plants that experienced a 6 d flood can utilize post-flood applied N to induce yield gains (Table 5). Total soil NO_3_-N concentration measured at 14 DAFI was a reasonably precise predictor of ear-leaf N concentration at R1 and grain yield in 2018 and may have potential for use in making N application decisions post-flood compared to existing sampling options for plant N status. The use of leaf greenness values may serve as an approximation of total soil NO_3_-N concentration, but increased accuracy beyond the level observed in the current research is likely needed to be effective for management. Scharf et al. [28] also observed limitations on using relative SPAD values to predict yield response when collected on the date of N application. Another challenge with using leaf greenness is that these values can vary by hybrid (independent of N status), which may limit the utility of SPAD to predict crop N need post-flood [29]. Producers from areas with high rainfall in the spring should consider reducing pre-plant N applications and employing post-flood N applications at mid-vegetative stages with conventional equipment (if crop height allows) or high-clearance equipment to ensure that adequate soil NO_3_-N for grain production is available after flooded conditions subside.

## 4. Materials and Methods

### 4.1. Study Sites and Experimental Design

A field experiment was conducted at the Waterman Agricultural and Natural Resources Laboratory (WNRL) (40°00’ N 83°02’ W, 200m elevation) in Columbus, OH, for two years (2017 and 2018) and at the Western Agricultural Research Station (WARS) in South Charleston, OH (39°51’ N 83°39’ W; 340 m elevation) in 2018 only. At WNRL, the soil type was a Crosby (fine, mixed, active, mesic Aeric Epiqualf) silt loam and a Celina (fine, mixed, active, mesic Aquic Hapludalf) silt loam in 2017 and 2018, respectively. The soil at WARS in 2018 was a Strawn (fine-loamy, mixed, active, mesic Typic Hapludalf)-Crosby silt loam. The previous crop at WNRL and WARS was soybean (*Glycine max* (L.) Merr.) and pumpkin (*Cucurbita pepo* L.), respectively. A composite (eight cores) soil sample to a depth of 20 cm was collected prior to planting for each field and analyzed for soil nutrient properties (Table 7). Monthly and daily weather data were compiled using the Ohio Agricultural Research and Development Center (OARDC) Weather Station Network located at both WNRL and WARS [30].

The experiment was conducted using a split-plot randomized complete block design with four replications (blocks). The whole-plot factor was flood duration, or FD (0, 2, 4 or 6 days), and the subplot factor was N management practice, or NMP (0 kg N ha^−1^, 134 kg N ha^−1^ applied pre-plant, or 134 pre-plant + 67 kg N ha^−1^ applied post-flood). Each subplot at WNRL was 3.1 × 7.6 m (four rows, 76 cm spacing) and 3.1 × 6.1 m in 2017 and 2018, respectively. Subplot size was 3.1 × 12.3 m at WARS in 2018. Whole plots were separated spatially in the field by 3.1 m in all directions. The 0 kg N ha^−1^ treatment was used as a comparison to observe how different N management practices (pre-plant alone, pre-plant + post-flood) compared to a non-fertilized practice to determine if these N applications differentially impacted plant response in the event of differing FD. The 134 or 134 + 67 kg N ha^−1^ treatments were chosen to simulate a split-application practice where the producer applies N pre-plant with the intent to sidedress apply the remaining N later in the season. Aside from the 0 kg N ha^−1^ sub-plots, 134 kg N ha^−1^ was broadcast using a 3.1 m drop spreader (Gandy, Owatonna, MN, USA) incorporated using a disk (10 cm depth) to each sub-plot within 24 hours prior to planting as urea (46–0–0).

The field at WNRL was planted on 8 June 2017 and on 1 May in 2018, and the field at WARS was planted on 9 May 2018. The hybrid brand product P1443AM (Dupont Pioneer, Johnston, IA, USA) was planted at a seeding rate of 84,000 plants ha^−1^. Plots were planted using a four-row John Deere 7000 Max-Emerge (Moline, IL) planter at WNRL each year, and a Kinze 2000 (Williamsburg, IA) planter at WARS. Soil berms 10 cm in height were created around each whole plot after planting using a modified moldboard plow to minimize treatment influence on other plots and to ensure flood conditions were achieved after planting was completed [9]. Pests were managed during the season following standard agronomic practices [31]. Stand counts were assessed at V4 on all rows prior to flood initiation.

The flood treatments were initiated at the V4 growth stage on 27 June 2017 and 25 May 2018 at WNRL and on 31 May 2018 at WARS (262–278 growing degree days from planting to flood initiation, base 10 °C). The V4 growth stage was used as the starting stage as this coincides to the start of sidedress applications in this production environment. A drip irrigation line was laid adjacent to each row requiring flooded conditions. During flooding, each environment maintained similar irrigation flow rates (21–22 cm irrigation applied per 24 h period). This level of irrigation (plus ambient rainfall) was sufficient to maintain waterlogged conditions of 1 to 2 cm standing water on the soil surface in each main plot and has been cited as a depth to induce flooding in previous research [32]. Each drip irrigation line was regulated with a control valve, and each line in the whole plot was shut off at the completion of its respective FD. For the 134 + 67 kg N ha^−1^ treatments, the 67 kg N ha^−1^ was broadcast applied using a hand broadcast spreader (Handy Green II, Scotts, Marysville, OH) as urea seven days after the FD was terminated for each whole-plot treatment (7, 9, 11, and 13 DAFI for the 0, 2, 4, and 6 d FD, respectively) to minimize compaction issues that could stem from equipment use under wet soil conditions, and to reduce the likelihood of volatilization of the broadcast urea post-flood.

### 4.2. Plant Growth and Development, Total Soil Nitrate-N, and Grain Yield Measurements

Corn growth stage was determined on two plants per plot at 0 and 14 DAFI. Height (using the extended leaf method) and leaf greenness of the uppermost collared leaf (quantified with a SPAD 502c- meter, Konica Minolta, Ramsey, NJ, USA) were measured at 0, 14 and 21 DAFI (two plants per plot). At R1, soil samples were collected (20 cm depth, 10 cores per plot) and analyzed for total soil NO_3_-N [33]. In 2018, additional soil samples were collected (20 cm depth, 6 cores per plot) and analyzed for total soil NO_3_-N at 0 and 14 DAFI. Ear-leaf samples were collected (10 leaves per plot) from the center two rows at the R1 growth stage and analyzed for tissue N concentration using the total combustion method.

In 2018, additional plants were harvested (two per plot) at 0 and 14 DAFI to quantify shoot and root biomass, in addition to root length, area, and porosity (as a proxy for root cortical aerenchyma formation). The aboveground biomass (separated at the lowest node above the roots) was dried for 5 to 7 days at 55 °C and weighed. The root porosity was measured within 4 h of removal from the soil using the procedure described by Thomson et al. [34]. The root fresh weight was recorded, and then the roots were suspended in a wire cage below the scale and weighed in water. Initially, the volume of the root was quantified using Equation (1):

(1) $$V_{root}=\;\frac{RW_{air}-RW_{water}}{D_{water}}$$

where *V_root_* = volume of root, *RW_air_* = weight of root in air, *RW_water_* = weight of root in water, and *D_water_* = density of water. The temperature of the water was collected on each measurement date to adjust the density of water. The roots were then placed in second container of water, and the container was placed in a vacuum infiltration chamber. A vacuum pressure (145 kPa) was applied for three periods lasting 30 seconds each. After the vacuum infiltration process, the roots were returned to the wire cage and weighed suspended in water. The volume of gas in the root was quantified using Equation (2):

(2) $$V_{g\;root}=\;\frac{\left(RW_{vacuum}-RW_{water}\right)}{D_{water}}$$

where *V_g root_* = volume of gas in root, *RW_vacuum_* = weight of vacuumed root, *RW_water_* = weight of root in water, and *D_water_* = density of water. Porosity was then calculated using Equation (3):

(3) $$\%\;Porosity=\;\frac{\left(V_{g\;root}*100\right)}{V_{root}}$$

where *V_g root_* = volume of gas in root, and *V_root_* = volume of root. 

The roots were transferred to plastic bags and stored at 4 °C until measured for total root area (cm^2^) and total root length (cm) using the program Winrhizo (Regent Instruments Inc, Quebec, Canada). Roots were scanned using an HP Scanjet 4850 scanner (HP Development Company, L.P. Palo Alto, CA, USA). After root porosity, area, and length assessment, the roots were dried for 7 days at 55 °C and weighed.

Before harvest, final stands were measured and stalk lodging (stalk breakage below the primary ear) was evaluated by counting the number of plants lodged in the center two rows of each subplot. Corn was harvested on 9 November 2017 and on 27 September 2018 at WNRL by hand from 3.1 m of the center two rows. Each ear was shelled using a mechanical sheller (SCS-2 corn sheller, Agriculex Inc. Guelf, Ontario, Canada), and moisture was quantified using a grain moisture meter (mini GAC grain moisture analyzer, Dickey-John, Auburn, IL, USA). Harvest was conducted on 3 October 2018 at WARS on the entire length of the center two rows using an 8-XP plot combine (Kincaid, Haven, KS, USA) and grain gauge with a moisture tester (HarvestMaster, Logan, UT, USA). Reported yields have been adjusted to 155 g kg^−1^ grain moisture.

NUE (yield gain in kg per unit N applied in kg) was calculated within each FD as the yield gained over the previous level of N application divided by the total additional units applied. For NUE of the 134 NMP, the yield gained by applying 134 kg N ha^−1^ over 0 kg N ha^−1^ was divided by 134. The NUE of the 134 + 67 NMP was calculated as the yield gained by applying 134 + 67 kg N ha^−1^ over 134 kg N ha^−1^ only divided by 67.

### 4.3. Statistical Analysis

To determine treatment effects on vegetative stage, leaf greenness, height, biomass, root characteristics, and available soil NO_3_-N, analysis of variance was conducted within each measurement date using the GLIMMIX procedure in SAS 9.4. (SAS Institute, Cary, NC, USA). Data were averaged across plants when the sampling unit (plant) was smaller than the experimental unit (plot) prior to analysis. Each site-year (SY), FD, NMP, and all interactions were treated as fixed effects. The random effects were replication (rep) nested in SY and FD × rep nested in SY (defined error term for whole-plot factor). When the F-test was significant for a model parameter (alpha = 0.05), means were separated using the LSMEANS statement. Due to the minimal significant interactions between SY and FD, NMP, and FD × NMP, the data were pooled across SY for presentation. Any observed interactions between SY and fixed factors were due to differences in magnitude, with treatments exhibiting similar rank order in each SY. To assess the relationship of yield and FD, the REG procedure was used to assess yield loss per day of flooding using a linear model.

A partial economic budget analysis was conducted to evaluate partial return for each treatment using the returns from marketing yield at 155 g kg^−1^. The price of corn used was $0.14 kg^−1^. The price of N and the cost of each N application was $0.81 kg^−1^ [35] and $14.36 ha^−1^ [36], respectively. Linear–plateau regression models to describe ear-leaf N concentration, yield, and leaf greenness responses to total soil NO_3_-N in 2018 were determined using the NLIN procedure in SAS 9.4.

## Figures and Tables

**Table 1:** Average monthly temperature and monthly cumulative precipitation and the monthly 30-year averages (1989–2018) for the Waterman Agricultural and Natural Resources Laboratory (WNRL) in 2017 and 2018, and the Western Agricultural Research Station (WARS) in 2018.

**WNRL Average Monthly Temperature (°C)**						
Year	May	June	July	Aug	Sept	Oct
2017	17.2	22.4	23.7	22.2	19.8	14.9
2018	21.9	23.2	24.0	23.7	21.4	13.4
30-year	17.1	22.1	23.6	22.7	19.0	12.5
**WNRL Total Monthly Precipitation (mm)**						
Year	May	June	July	Aug	Sept	Oct
2017	130.6	100.3	220.0	42.4	56.6	106.7
2018	120.4	179.3	74.7	137.4	69.1	53.3
30-year	110.2	112.9	122.5	86.1	79.0	69.0
**WARS Average Monthly Temperature (°C)**						
Year	May	June	July	Aug	Sept	Oct
2018	21.0	22.4	22.8	22.7	20.7	12.8
30-year	16.9	21.6	23.0	22.0	18.3	12.0
**WARS Total Monthly Precipitation (mm)**						
Year	May	June	July	Aug	Sept	Oct
2018	71.1	115.3	94.2	93.2	157.0	35.8
30-year	104.4	99.8	120.4	68.1	79.8	71.6

**Table 2:** Daily maximum temperature, daily average temperature, and daily precipitation from 0 days after flood initiation (DAFI) through 14 DAFI at the Waterman Agricultural and Natural Resources Laboratory (WNRL) in 2017 and 2018, and the Western Agricultural Research Station (WARS) in 2018.

	WNRL, 2017			WNRL, 2018			WARS, 2018		
DAFI	Max Temp	Avg Temp	Precip	Max Temp	Avg Temp	Precip	Max Temp	Avg Temp	Precip
day	°C		mm	°C		mm	°C		mm
0	22.8	17.2	0.0	30.9	23.6	0.0	30.3	19.0	3.3
1	26.2	19.6	0.0	30.2	23.1	27.7	28.5	17.1	5.1
2	30.8	24.2	0.0	33.8	25.9	0.0	28	15.7	0.0
3	29.0	24.9	7.4	32.5	26.9	0.0	29.3	20.4	0.5
4	28.4	23.8	12.2	31.6	26.8	0.0	26	22.5	0.0
5	29.6	24.9	0.0	30.1	25.7	11.2	22.9	22.3	2.8
6	31.6	25.1	0.0	31.2	25.4	0.0	21	21.9	0.3
7	29.8	24.9	0.0	28.6	22.6	22.1	29.3	20.9	0.0
8	30.6	25.1	0.0	28.3	22.9	0.0	31.9	21.6	16.0
9	26.5	23.4	12.4	28.5	23.2	1.5	31.1	23.0	2.8
10	28.8	21.5	22.6	24.5	20.2	0.0	26.8	20.7	9.1
11	27.0	21.9	0.3	19.9	17.1	16.8	24.3	22.4	4.8
12	26.7	21.1	0.0	22.0	15.8	0.0	27.3	24.7	8.1
13	28.2	21.7	47.8	28.3	20.1	0.0	29.4	27.6	12.2
14	29.5	24.5	8.1	29.0	21.8	7.5	28.6	28.0	0.3
Average	28.3	22.9	7.4	28.6	22.7	6.5	27.6	21.9	4.4

Avg—average; Precip—precipitation; Temp—temperature.

**Table 3:** Root length, area, and porosity for each flood duration (FD) and nitrogen management practice (NMP) 0, 7, and 14 days after flood initiation (DAFI). *p* values are presented within each column for FD, NMP, and FD × NMP. Different letters indicate differences in means within each column and FD × NMP interaction.

FD	NMP	Shoot Biomass		Root Biomass		Root Length		Root Area		Root Porosity	
		0 DAFI	14 DAFI	0 DAFI	14 DAFI	0 DAFI	14 DAFI	0 DAFI	14 DAFI	0 DAFI	14 DAFI
days	kg N ha^−1^	g plant^−1^				cm		cm^2^		%	
0	0	2.27	18.7	0.28	2.24	134.8	303.8	13.47	18.10 ^d^	5.88	6.23
	134	2.20	22.3	0.29	2.61	143.7	315.9	13.51	18.84 ^bcd^	5.95	6.64
	134 + 67	2.18	20.7	0.27	2.62	129.3	312.0	12.6	19.20 ^bcd^	5.87	6.36
2	0	2.28	12.8	0.27	1.74	135.2	330.0	14.10	19.38 ^bcd^	5.69	8.70
	134	1.87	16.3	0.25	1.92	135.2	303.4	14.11	18.56 ^cd^	5.85	8.44
	134+67	2.30	17.4	0.31	2.52	143.0	340.2	13.56	20.11 ^abc^	6.19	7.64
4	0	2.03	10.9	0.29	2.21	131.0	349.3	13.53	20.57 ^ab^	5.93	9.12
	134	2.39	13.5	0.31	2.79	136.2	356.5	13.92	20.44 ^ab^	6.00	8.33
	134 + 67	2.10	12.1	.031	2.19	146.9	320.6	13.92	18.75 ^cd^	5.43	8.42
6	0	2.15	11.4	0.25	2.50	132.4	382.0	13.08	21.46 ^a^	5.17	10.55
	134	2.42	12.0	0.29	2.58	115.2	332.7	12.27	20.21 ^abc^	5.81	8.92
	134 + 67	2.28	10.3	0.28	2.33	138.0	348.9	13.81	19.94 ^abc^	6.71	9.35
	FD	0.9016	<0.0001	0.4370	0.1667	0.7091	0.1237	0.2991	0.0512	0.9963	0.0275
	NMP	0.9442	0.0361	0.2856	0.1507	0.4776	0.3794	0.9685	0.5872	0.7634	0.2739
	FD × NMP	0.2026	0.5434	0.2231	0.1593	0.4460	0.1078	0.3464	0.0371	0.8849	0.7707

**Table 4:** Ear-leaf N concentration and total soil nitrate-nitrogen (NO_3_-N) collected at R1, corn grain yield, and partial economic return for the interaction of each flood duration (FD) and nitrogen management practice (NMP). *p* values are presented within each column for FD, NMP, and FD × NMP. Different letters within a column indicate differences in means for the FD × NMP interaction.

FD	NMP	Ear-Leaf N Concentration	Total Soil NO_3_-N	Corn Grain Yield	Partial Return
Days	kg N ha^−1^	g kg^−1^		kg ha^−1^	$ ha^−1^
0	0	23.2 ^e^	2.0 ^b^	8684 ^d^	1283 ^c^
	134	30.7 ^ab^	5.1 ^b^	14315 ^ab^	1981 ^a^
	134 + 67	32.2 ^a^	12.0 ^a^	15301 ^a^	2054 ^a^
2	0	20.2 ^f^	1.8 ^b^	6435 ^ef^	951 ^de^
	134	25.4 ^d^	2.3 ^b^	10839 ^c^	1468 ^c^
	134 + 67	29.8 ^bc^	4.1 ^b^	13810 ^ab^	1834 ^ab^
4	0	19.0 ^f^	1.9 ^b^	5324 ^f^	787 ^e^
	134	22.7 ^e^	2.2 ^b^	7589 ^de^	988 ^d^
	134 + 67	29.5 ^bc^	3.5 ^b^	12938 ^b^	1705 ^b^
6	0	20.3 ^f^	1.9 ^b^	5684 ^f^	840 ^de^
	134	22.3 ^e^	2.0 ^b^	6618 ^ef^	845 ^de^
	134 + 67	28.0 ^c^	2.5 ^b^	10262 ^c^	1309 ^c^
	FD	<0.0001	0.0002	<0.0001	<0.0001
	NMP	<0.0001	0.0003	<0.0001	<0.0001
	FD × NMP	0.0001	0.0091	<0.0001	<0.0001

**Table 5:** Nitrogen use efficiency (NUE) for each flood duration (FD). The NUE was measured as the yield gained per unit N applied over the previous level of nitrogen management practice (NMP) (yield gained by applying 134 kg N ha^−1^ over 0 kg N ha^−1^ or yield gained by applying 134 + 67 kg N ha^−1^ over 134 kg N ha^−1^ only). *p* values are presented within each column for FD. Different letters indicate differences in means for each FD within each column.

FD	NUE of NMP	
	134	134 + 67
Days	kg yield kg N^−1^	
0	40.67 a	14.09 c
2	30.69 ab	42.54 bc
4	18.22 bc	79.83 a
6	5.31 c	52.48 ab
FD	0.0011	0.0011

**Table 6:** Interaction of flood duration (FD) and nitrogen application (NMP) for ear-leaf N concentration at R1, total soil nitrate-nitrogen (NO_3_-N) concentration 0 and 14 days after flood initiation (DAFI), and leaf greenness from 14 and 21 DAFI in 2018. *p* values are presented within each column for FD, NMP, and FD × NMP. When present, different lowercase letters indicate differences in means within each column and FD × NMP interaction and different uppercase letters denote differences in NMP.

FD	NMP	Ear-Leaf N	Total Soil NO_3_-N		Leaf Greenness	
			0 DAFI	14 DAFI	14 DAFI	21 DAFI
days	kg N ha^−1^	g kg^−1^	mg kg^−1^		SPAD	
0	0	22.6 ^de^	16.8 ^B^	10.5 ^efg^	46.9	48.8 ^c^
	134	30.1 ^ab^	40.8 ^A^	28.1 ^b^	51.6	54.6 ^a^
	201	31.3 ^a^	38.5 ^A^	47.8 ^a^	50.6	52.7 ^ab^
2	0	19.2 ^g^	13.5 ^B^	6.7 ^g^	36.6	37.7 ^ef^
	134	24.5 ^d^	43.8 ^A^	14.8 ^def^	44.4	48.8 ^bc^
	201	29.1 ^bc^	44.0 ^A^	25.1 ^bc^	45.7	49.1 ^bc^
4	0	18.7 ^g^	16.5 ^B^	3.8 ^g^	31.4	32.0 ^g^
	134	21.8 ^ef^	38.6 ^A^	8.0 ^fg^	39.5	40.2 de
	201	28.7 ^bc^	50.3 ^A^	22.0 ^bcd^	39.7	48.3 ^c^
6	0	20.5 ^fg^	16.9 ^B^	4.6 g	30.0	31.7
	134	22.4 ^def^	40.8 ^A^	6.5 ^g^	32.7	35.6 ^fg^
	201	27.9 ^c^	41.8 ^A^	18.1 ^cde^	33.7	42.9 ^d^
	FD	<0.0001	0.7368	<0.0001	<0.0001	<0.0001
	NMP	<0.0001	<0.0001	<0.0001	<0.0001	<0.0001
	FD × NMP	0.0018	0.2519	0.0006	0.1882	0.0003

**Table 7:** Soil sample analysis for the Waterman Agricultural and Natural Resource Laboratory (WNRL) in 2017 and 2018, and the Western Agricultural Research Station (WARS) in 2018.

Year	Site	Organic Matter (g kg^−1^)	Cation Exchange Capacity (cmol_c_ kg^−1^)	pH	P, Mehlich-3	Exchangeable Cations, Mehlich-3		
						K	Mg	Ca
					(mg kg^−1^)			
2017	WNRL	28	12.6	7.2	155	213	371	2350
2018	WNRL	27	10.4	7.0	61	158	327	1868
	WARS	29	15.4	6.5	136	140	596	2323

## Supplementary Materials

The following are available online at https://www.mdpi.com/2223-7747/9/3/348/s1, Table S1: Average vegetative growth stage, plant height, and leaf greenness (SPAD) at the day of flood initiation for each flood duration (FD) and N management practice (NMP). *p* values are presented within each column for FD, NMP, and FD × NMP., Table S2: Grain moisture at harvest and stalk lodging for each flood duration (FD) and N management practice (NMP). *p* values are presented within each column for FD, NMP, and FD × NMP., Table S3: Biomass of 10 ear leaves collected at the R1 growth stage. *p* values are presented within each column for FD, NMP, and FD × NMP. Different letters denote differences in means for the FD x NMP interaction.
