# Supplementary material for: Grain Yield Response of Corn (Zea mays L.) to Nitrogen Management Practices and Flooding
Source: Plants (Basel). 2020 Mar 10;9(3):348. doi: 10.3390/plants9030348 (PMC7154854; doi:10.3390/plants9030348)
Supplement: Supplementary file 1 [file plants-09-00348-s001.pdf]

Table S1. Average vegetative growth stage, plant height, and leaf greenness (SPAD) at the day of flood initiation for each flood duration (FD) and N management practice (NMP). *p* values are presented within each column for FD, NMP, and FD × NMP.

FD	NMP	Growth Stage	Plant Height	Leaf Greenness
days	kg N ha^{-1}		cm	SPAD
0		4.2	43.1	39.3
2		4.3	43.2	39.2
4		4.3	44.2	38.6
6		4.3	44.2	39.9
	0	4.3	43.4	39.3
	134	4.3	43.6	39.2
	134+67	4.3	43.9	39.3
	FD	0.8315	0.4873	0.2994
	NMP	0.9432	0.4942	0.9920
	FD × NMP	0.0605	0.1133	0.0527

Table S2. Grain moisture at harvest and stalk lodging for each flood duration (FD) and N management practice (NMP). *p* values are presented within each column for FD, NMP, and FD × NMP.

FD	NMP	Grain Moisture	Stalk Lodging
days	kg N ha^{-1}	%	
0		19.6	0.2
2		20.0	1.1
4		20.0	0.1
6		20.3	0.3
	0	20.1	0.1
	134	19.9	0.9
	134+67	19.9	0.3
	FD	0.3779	0.2038
	NMP	0.8907	0.0604
	FD × NMP	0.9598	0.0941

Table S3. Biomass of 10 ear leaves collected at the R1 growth stage. *p* values are presented within each column for FD, NMP, and FD × NMP. Different letters denote differences in means for the FD x NMP interaction.

**FD**	**NMP**	**Ear Leaf Biomass**
**days**	**kg N ha^{-1}**	**g**
0	0	40.0 ^{c}
	134	44.4 ^{b}
	134+67	49.5 ^{a}
2	0	31.2 ^{d}
	134	41.4 ^{bc}
	134+67	44.5 ^{b}
4	0	23.1 ^{e}
	134	31.6 ^{d}
	134+67	41.2 ^{bc}
6	0	21.3 ^{e}
	134	24.9 ^{e}
4	134+67	33.9 ^{d}
	FD	<0.0001
	NMP	<0.0001
	FD × NMP	0.0176
